# *In Vivo* Venous Assessment of Red Blood Cell Aggregate Sizes in Diabetic Patients with a Quantitative Cellular Ultrasound Imaging Method: Proof of Concept

**DOI:** 10.1371/journal.pone.0124712

**Published:** 2015-04-23

**Authors:** Julien Tripette, Linh-Chi Nguyen, Louise Allard, Pierre Robillard, Gilles Soulez, Guy Cloutier

**Affiliations:** 1 Laboratory of Biorheology and Medical Ultrasonics, Research Center, University of Montreal Hospital (CRCHUM), Québec, Canada; 2 Institute of Biomedical Engineering, University of Montreal, Québec, Canada; 3 Department of Radiology, University of Montreal Hospital (CHUM), Québec, Canada; 4 Department of Radiology, Radio-Oncology and Nuclear Medicine, University of Montreal, Québec, Canada; Baylor College of Medicine, UNITED STATES

## Abstract

**Background:**

Diabetic patients present higher level of red blood cell (RBC) aggregation contributing to the development of vascular complications. While it has been suggested that this hematology/rheology parameter could bring additional prognostic information for the management of those patients, RBC aggregation screening is not included as a clinical practice. Most medical centers are not equipped to measure properly this parameter, although sedimentation tests can bring some indication. Here, we aimed at evaluating the feasibility of using ultrasound to assess *in-vivo *hyper-aggregation in type 2 diabetic patients.

**Research design and methods:**

Seventeen diabetic patients and 15 control subjects underwent ultrasound measurements of RBC aggregation in both cephalic and great saphenous veins. Non-invasive *in-vivo* ultrasound measurements were performed using a newly developed cellular imaging technique, the structure factor size and attenuation estimator (SFSAE). Comparisons with an *ex-vivo *gold standard rheometry technique were done, along with measurements of pro-aggregating plasma molecule concentrations.

**Results:**

*In-vivo* RBC aggregation was significantly higher in diabetic patients compared with controls for cephalic vein measurements, while a trend (*p* = 0.055) was noticed in the great saphenous vein. SFSAE measurements were correlated with gold standard *in-vitro *measures, fibrinogen and C-reactive protein plasma concentrations.

**Conclusion:**

RBC aggregation can be measured *in-vivo* in diabetic patients using ultrasound. Prospective studies are needed to determine whether the SFSAE method could help clinicians in the early management of vascular complications in this patient population.

## Introduction

Red blood cell (RBC) aggregation is a normal and reversible phenomenon influencing blood flow throughout the circulation. The basic structure of RBC aggregates (or “rouleaux”) is an alignment of several cells, very similar to a “stack of coins” [1]. RBC aggregates are likely to form in venules and veins, where flow shear forces are low, and they are dispersed in regions with more important hemodynamic stress. Despite RBC aggregation is considered as a normal phenomenon, hyper-aggregation (which describes bigger aggregates in the form of clumps) is potentially deleterious in a wide range of pathologies, including diabetes, as it impedes blood circulation and decreases tissue perfusion [2, 3].

While the exact mechanisms are still not clearly known, RBC hyper-aggregation is usually attributed to some changes in the plasma concentration of fibrinogen (Fb) and other inflammatory acute proteins (*e*.*g*., immunoglobulin G, haptoglobin, C-reactive protein) [4–7], which modify the interaction between RBC [8]. RBC hyper-aggregation affects flow dynamics and RBC distribution in the microvascular network (*e*.*g*., microvessel plugging and shunting), promoting long term impairments of the vasomotor control [9]. For these reasons, hyper-aggregation has been thought to participate in the development of vascular complications such as diabetic angiopathies [1, 10], and some authors suggested that its measure could bring early prognostic information for the management of vascular complications in those patients [11–13].

Despite the fact that several *in-vitro* laboratory techniques can be used to measure RBC aggregation (including blood smear microscopic observation, sedimentation rate, low shear viscosity measurement, laser-assisted optical methods, *etc…*), they all present some limitations and require blood sampling. The current gold standard method is a laser-assisted scattering technique associated with a Couette flow system, which is able to perform *ex-vivo* measurements of RBC aggregation kinetics and adhesion forces of aggregates [14, 15]. While this method is able to provide a complete rheological profile of the patient’s blood sample (*i*.*e*., aggregation time and aggregate strength), it is still unavailable in most clinical centers and cannot be used to measure RBC aggregation in real time at the bedside.

The present study proposes a new cellular ultrasound imaging technique, the structure factor size and attenuation estimator (SFSAE) [16–18], to estimate *in-vivo* RBC aggregate sizes in diabetic patients non-invasively and in real time. The aim of this paper is to test the hypothesis that the SFSAE technique can be used to detect diabetes-related hyper-aggregation *in-situ* within superficial veins accessible for ultrasound monitoring purpose.

## Methods

### Population and protocol

Seventeen diabetic patients (DIAB group) and 15 healthy subjects (CONT group) participated in the study. Diabetic patients had all been diagnosed with type 2 diabetes mellitus for more than 5 years and had oral medication to regulate their glycaemia. Both DIAB and CONT groups were matched in age (53.4 ± 1.7 years *vs*. 55.2 ± 2.3 years) and gender (6F and 9M vs. 8F and 9M). However, participants presented different average body mass indexes (BMI) (31.4 ± 1.3 *vs*. 24.2 ± 0.9 kg/m^2^ for DIAB and CONT, respectively). Among diabetic patients, 10 subjects were diagnosed with Rutherford stage 1 or 2 lower limb peripheral arterial disease [19]. All subjects were nonsmokers and provided a written informed consent. The human ethic committee of the University of Montreal Hospital approved the study.

Recruited subjects came at the Department of Radiology of the University of Montreal Hospital for data collection. After an overnight fast, 30 mL of venous blood was drawn using EDTA, sodium-citrated and dry vacutained tubes. Blood analyses were performed within two hours after collection. Ultrasound measurements of RBC aggregation began about 30 min after subjects took a breakfast.

### Biochemical analyses

Plasma concentrations of glycated hemoglobin (HbA1c; G7 HPLC analyzer, Tosoh Bioscience Inc., South San Francisco, California), fibrinogen (BCS XP System, Siemens Healthcare, Erlangen, Germany), immunoglobulin G (IgG; BN II System, Siemens Healthcare), haptoglobin (Hp; BN II System, Siemens Healthcare) and C-reactive protein (CRP; BN II System, Siemens Healthcare) were measured from blood samples with standard laboratory tests.

### Hematocrit and ex-vivo RBC aggregation measurements

The hematocrit (Hct) was measured by micro-centrifugation. RBC aggregation time (T_A_), RBC aggregation kinetics (S_10_, which reflects the amplitude of the aggregation phenomenon), and the disaggregation threshold (*γ*_thr_, which reflects aggregate strength) were determined using an *ex-vivo* laser-based laboratory instrument (Regulest, Florange, France). This method based on a Couette flow formed by two concentric cylinders allows applying shear forces to the blood sample to assess aggregation indices under different hemodynamic conditions. To date, this method is still considered as the gold-standard to measure RBC aggregation in human subjects [14]. Blood sampled in EDTA tubes were used to perform these tests. All measurements were done at 37°C.

### In-vivo RBC aggregation assessment

**Fig 1:**
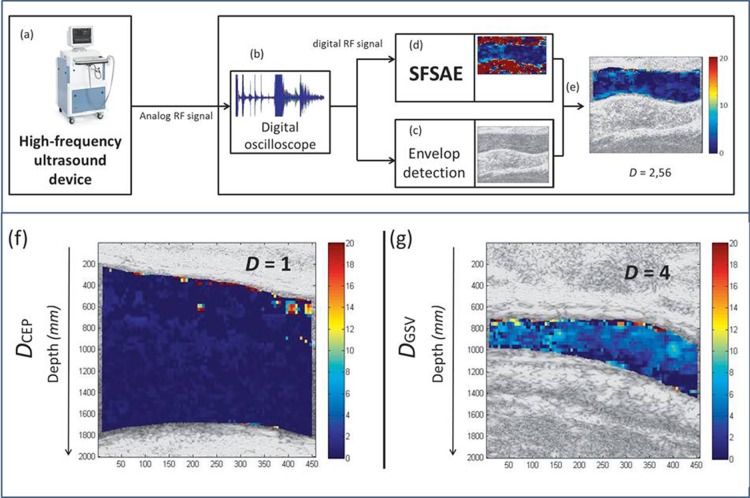
Top: view of the experimental set-up. (a) Raw radio-frequency (RF) data are acquired with a high-frequency ultrasound device (Vevo 770, Visualsonics, Toronto, Canada). (b) Analog RF data are digitalized and recorded on a computer. (c) B-mode images can be reconstructed for display purpose, and (d) a parametric image of *D* representing local level of red blood cell (RBC) aggregation can be computed with the SFSAE cellular imaging method. (e) The parametric image is superimposed on the B-mode image after segmentation and calculation of the *D* index. Finally, local *D* values temporally and spatially averaged are computed for diagnosis. Bottom: example of images obtained at different levels of aggregation. (f) Low level of RBC aggregation in a control cephalic vein. (g) High level of RBC aggregation in a diabetic great saphenous vein.

Subjects were in supine position during the whole examination. The cephalic vein in the proximal portion of the forearm (CEP) and the great saphenous vein in the distal portion of the leg (GSV) were scanned with a high frequency ultrasound system (Vevo 770, Visualsonics, Toronto, Canada) equipped with a mono element oscillating probe (RMS-710 transducer, central frequency of 25 MHz). A new cellular imaging mode based on the SFSAE was employed to characterize RBC aggregation, as already used *in-vivo* in animal models [17, 18]. Venous monitoring was preferred because low shear rates offer favorable conditions for the formation and maintenance of aggregates. The SFSAE utilizes a spectral model that allows extracting the mean aggregate diameter *D*, from the analysis of radio-frequency (RF) echoes backscattered by blood. *D* is the ratio of the diameter of a fractal isotropic aggregate to the diameter of one RBC, and it therefore increases proportionally with RBC aggregation. A value of *D* < or = 1 indicates disaggregated RBC [20]. The SFSAE model compensates for skin and tissue attenuations allowing *D* to be independent of subject adiposity. RF ultrasound echoes from longitudinal views of each vessel were acquired using a Panametrics receiver (5900 PR, Waltham, MA, USA) and a digital oscilloscope (Gagescope 8500CS, Montreal, QC, Canada), as described in Fig 1A. The RF data corresponds to the raw ultrasound signal that has not been modified by any signal processing normally used to obtain classical B-mode images. The proposed cellular imaging technique is described in details elsewhere [20, 21]. For each subject, a sequence of 25 consecutive vascular images of *D* was spatially averaged in both the CEP (*D*_CEP_) and the GSV (*D*_GSV_). A schematic presentation of the experimental setting is described in Fig 1B–1E. This new imaging technique has already been validated under *in-vitro* and *in-vivo* conditions (not in human) [17, 18, 20–22].

### Flow shear rate measurement

Because RBC aggregation is dependent on the shear condition associated with the blood flow, we measured this potential confounding variable at the same locations as RBC aggregation measurements using a clinical ultrasound scanner (Aixplorer, Supersonic Imagine, France), as done elsewhere [18, 22]. B-mode images and Doppler mean velocities (*V*_*mean*_) were acquired in both CEP and GSV. *V*_*mean*_ was computed by time-averaging the maximum center line velocity measured in pulse-wave Doppler mode over a few cardiac cycles (typically 5). The vein diameter (*D*_*i*_) was measured from B-mode images. Then, the mean shear rate within the vein (γ) was calculated as [23]:

$$\gamma =\frac{2V_{mean}}{r}\left(\frac{n}{n+1}\right)$$

where *r* is the radius of the vessel and *n* is a constant related to the flow velocity profile. In this study, we supposed that velocity profiles in the veins were parabolic, which is consistent with steady Newtonian flow (*i*.e., *n* = 2). The presence of RBC aggregation likely blunted velocity profiles; estimated shear rates could thus be slightly overestimated.

For very low velocities in veins (*i*.*e*., *V*_*mean*_ < 0.6 cm/s), the L15-4-38 ultrasound probe (Supersonic Imaging, France) could not detect the time-varying velocity due to the wall filter (the wall filter is an instrument setting avoiding low velocity vibrations from surrounding tissues to corrupt the flow waveform). Since a velocity of 0 cm/s seems unlikely to occur in living veins, we assumed *V*_*mean*_ = 0.6 cm/s for all velocities not detected by the probe.

### Statistical analyses

Student *t*-tests were used to compare measures between groups. One-tail analyses were performed for blood markers as one-way variations were expected. Linear regressions and Pearson tests were performed to determine relations between *D*_CEP_ and *D*_GSV_, and between ultrasound RBC aggregation parameters and blood parameters (*i*.*e*., plasma proteins and *ex-vivo* measurements of RBC aggregation). Analyses were conducted using R (R.app GUI 1.40-devel, R Foundation for Statistical Computing, Vienna, Austria) and Sigma Stat (v. 3.1, San Jose, California, USA) packages. Significant differences referred to *p* < 0.05. Values are expressed as mean ± SEM.

## Results

### Biochemical analyses

As shown in Table 1, the DIAB group presented significantly higher HbA1c values compared to the CONT group (7.38 ± 0.29 *vs*. 5.62 ± 0.05%; *p* < 0.001). No significant difference was found between groups for IgG. Diabetic patients exhibited higher haptoglobin (CONT: 1.41 ± 0.18 vs. DIAB: 1.96 ± 0.20 g L^-1^; *p* = 0.026), CRP (CONT: 2.50 ± 0.85 vs. DIAB: 5.36 ± 0.92 mg L^-1^; *p* = 0.017) and fibrinogen concentrations (CONT: 2.92 ± 0.24 vs. DIAB: 3.93 ± 0.12 g L^-1^; *p* = 0.039).

**Table 1:** Subject characteristics and blood markers.

	DIAB group	CONT group
**Age (years)**	55.2 ± 2.3	53.4 ± 1.7
**Gender**	8 women / 9 men	6 women / 9 men
**BMI**	31.4 ± 1.3*	24.2 ± 0.9
**HbA1c (%)**	7.38 ± 0.29**	5.62 ± 0.9
**Hematocrit (%)**	42 ± 1	44 ± 1
**Fibrinogen (g/L)**	3.93 ± 0.12*	2.92 ± 0.24
**Immunoglobulin G (g/L)**	10.96 ± 0.58	10.12 ± 0.73
**Haptoglobin (g/L)**	1.96 ± 0.20*	1.41 ± 0.18
**C-reactive protein (mg/L)**	5.36 ± 0.92*	2.50 ± 0.85

BMI: body mass index, HbA1c: glycated hemoglobin.

* *p* < 0.05,

** *p* < 0.001, mean ± SEM.

### Hematocrit and ex-vivo measurements of RBC aggregation

**Fig 2:**
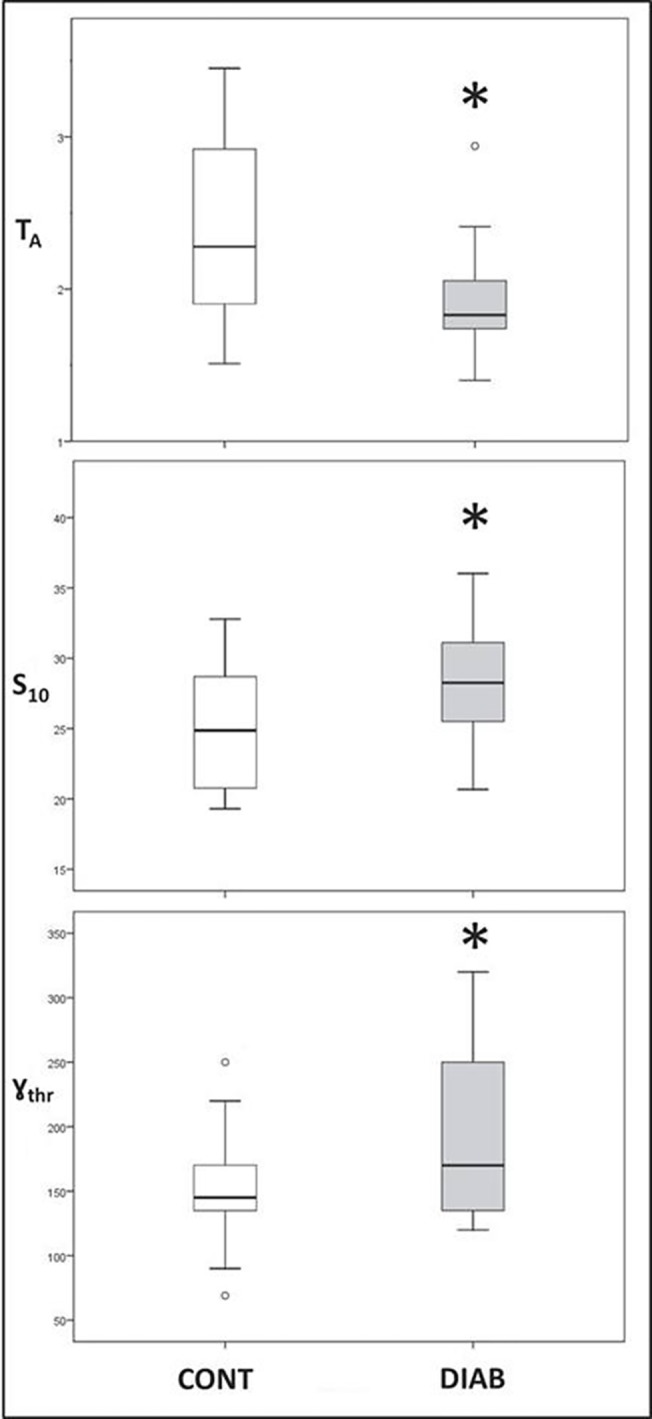
Comparison between control subjects (CONT) and diabetic patients (DIAB) for *ex-vivo* red blood cell aggregation parameters (T_A_: aggregation time in second; S_10_: RBC aggregation kinetics (no unit); *γ*_thr_: disaggregation threshold in second^-1^). Data are presented as Tukey boxplots. ○ describes outliers. * *p* < 0.05, mean ± SEM.

Hct values were statistically similar between groups (Table 1), whereas gold standard laser-assisted optical measurements indicated a higher RBC aggregation in the DIAB group (*i*.*e*., lower T_A_: 1.92 ± 0.10 *vs*. 2.41 ± 0.16 s in controls, *p* = 0.016; higher S_10_: 28.6 ± 1.1 *vs*. 25.1 ± 1.2 (no unit), *p* = 0.047; and higher *γ*_thr_: 193 ± 16 *vs*. 150 ± 12 s^-1^, *p* = 0.046, see Fig 2).

### In-vivo measurements of RBC aggregation and flow shear rates

**Fig 3:**
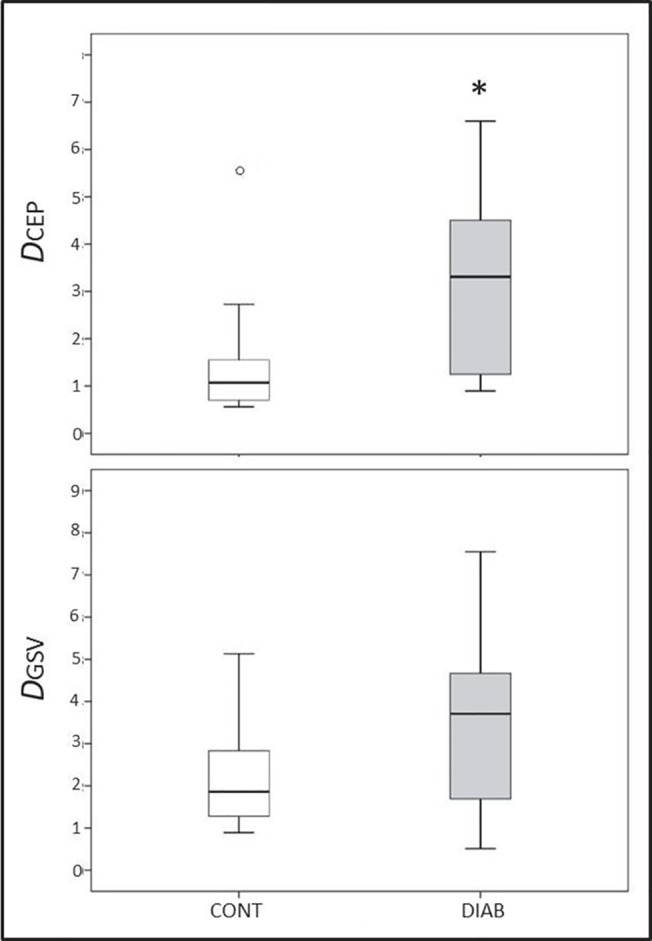
Comparison between control subjects (CONT) and diabetic patients (DIAB) for ultrasound *in-vivo* measurements of RBC aggregation in the cephalic vein CEP (*D*_CEP_) and in the great saphenous vein GSV (*D*_GSV_). Data are presented as Tukey boxplots. ○ describes outliers. * *p* < 0.05, mean ± SEM.

As presented in Fig 3, we observed higher ultrasound-based *in-vivo* and *in-situ* RBC aggregation in the CEP of diabetic patients (*D*_CEP_: 3.20 ± 0.44 vs. 1.49 ± 0.35 (no unit), *p* = 0.004). CEP shear rates measured with ultrasound were found higher in the DIAB group than in the CONT group (*p* = 0.024; Table 2). Because higher shear rates tend to disaggregate RBC, this is noticeable that we could nevertheless observe higher aggregation in diabetes. *D*_GSV_ exhibited a trend for higher RBC aggregation in the DIAB group (*D*_GSV_: 3.64 ± 0.49 vs. 2.30 ± 0.35 (no unit); *p* = 0.055). *In-situ* shear rates in that vessel were also significantly higher in the DIAB group compared to the CONT group (*p* = 0.018; Table 2). As also shown in that Table, no differences were found between groups for both CEP and GSV diameters. Flow velocities were higher in the DIAB group for both CEP and GSV (*p* = 0.020 and *p* = 0.030, respectively, see Table 2).

**Table 2:** Vessel diameters, flow velocities and shear rates.

	DIAB group	CONT group
**CEP mean diameter (mm)**	3.66 ± 0.26	4.03 ± 0.30
**CEP mean velocity (cm.s** ^**-1**^ **)**	10.5 ± 1.9*	4.9 ± 1.1
**CEP mean shear rates (s** ^**-1**^ **)**	85 ± 18*	34 ± 8
**GSV mean diameter (mm)**	2.60 ± 0.20	2.50 ± 0.10
**GSV mean velocity (cm.s** ^**-1**^ **)**	4.3 ± 0.5*	2.1 ± 0.7
**GSV mean shear rates (s** ^**-1**^ **)**	47 ± 7*	22 ± 6

* *p* < 0.05,

mean ± SEM.

### Correlations between in-vivo RBC aggregation and laboratory tests

A significant correlation was found between *D*_*CEP*_ and laser aggregometry *γ*_thr_ values (*r* = 0.47, *p* = 0.011), but not between *D*_GSV_ and *γ*_thr_ (*r* = 0.32, *p* = 0.11)_._ No significant correlation was noticed between *D*_CEP_ and other *ex-vivo* rheological parameters (T_A_ and S_10_). *D*_CEP_ correlated with fibrinogen (*r* = 0.54, *p* = 0.003) and CRP plasma concentrations (*r* = 0.53, *p* = 0.004). *D*_GSV_ exhibited a significant correlation with fibrinogen plasma concentrations as well (*r* = 0.43, *p* = 0.028).

## Discussion

In the present study, we observed higher levels of *in-vivo* RBC aggregation in diabetic patients compared to control subjects. Bigger aggregates were indeed imaged in the DIAB group under natural venous flow conditions. This study constitutes the first *in-vivo* quantitative assessment of RBC aggregate sizes in human subjects, and *a fortiori* in diabetic patients. These *in-vivo* results correlated with 1) the *ex-vivo* aggregate strength measurements and 2) pro-aggregating plasma molecule concentrations (*cf*. fibrinogen and CRP).

### Higher RBC aggregation in diabetic patients

No surprisingly, higher RBC aggregation was noted in diabetic patients compared to the control group. The *ex-vivo* gold standard laser-assisted optical assessments also showed higher RBC aggregation in the DIAB group. The lower aggregation time (*cf*. T_A_) and higher magnitude of S_10_ observed in this group indeed indicate that diabetic erythrocytes have a higher propensity to form aggregates. Moreover, the *ex-vivo* laser-assisted method showed stronger binding energy between aggregates of diabetic patients compared to control subjects. While these observations have been reported many times under *ex-vivo* conditions [10–12, 24], the present study confirmed for the first time that this phenomenon does actually exist *in-vivo*. In the cephalic vein, *D* was higher in diabetic patients indicating larger flowing aggregate sizes. We also noted a strong trend (*p* = 0.055) in the GSV for the same phenomenon.

### Characteristics of in-vivo RBC aggregation in diabetes mellitus

The correlation between *D*_CEP_ and *γ*_thr_ (*r* = 0.47, *p* = 0.011) indicates that the aggregate strength may be the most important determinant for observing *in-vivo* RBC aggregation (compared to other *ex-vivo* parameters reflecting the propensity to form aggregates: *i*.*e*., T_A_ and S_10_), at least in the diabetic population. While the interpretation of *in-vivo* RBC aggregation data traditionally involved analyses of indirect laboratory test indices: *e*.*g*., sedimentation rate, low-shear blood viscosity or laser aggregometry measures T_A_, S_10_ and *γ*_thr_ (or other similar indices depending on the manufacturer of the instrument) [15], our results suggest that future direct assessment of *in-situ* RBC aggregation is possible, especially in the diabetic population.

In the present study, *D*_CEP_ correlated with fibrinogen and CRP plasma concentrations (*r* = 0.54, *p* = 0.003; *r* = 0.53, *p* = 0.004, respectively), which are known promoters of RBC aggregation [5–7]. *D*_GSV_ exhibited a significant correlation with fibrinogen plasma concentration as well (*r* = 0.43, *p* = 0.028). The relation between high levels of fibrinogen and stronger RBC aggregates has already been shown [25], supporting the present association between high inflammatory acute-phase protein concentrations, elevated *ex-vivo* RBC aggregate strength, and higher *in-vivo* RBC aggregation in diabetic patients. The higher *in-vivo* RBC aggregation found concomitantly with higher shear rates (favoring the breaking down of reversible aggregates) in the diabetic population, for both cephalic and great saphenous veins, also support the hypothesis that enhanced RBC aggregating energies were determinant for explaining reported observations. The exact mechanism for the higher shear rate in veins of diabetic patients compared to the control population is unknown but might be associated with 1) a hemodynamic compensation phenomenon to avoid the deleterious effect of large flowing aggregates (inducing high blood viscosity) on the venous return, or 2) an arteriovenous shunting phenomenon that has already been described elsewhere [26]. We indeed observed higher mean velocities in the diabetic population (Table 2).

The validity of the SFSAE cellular imaging measures is supported by previous *ex-vivo* microscopic observations performed under dynamic conditions, which highlighted the spherical shape of compact aggregates in diabetes mellitus compared to normal cylindrical “rouleaux” observed in healthy subjects [27, 28]. Indeed, SFSAE images are based on a model assuming flowing RBC aggregates of spherical forms [16]. Therefore, from a physical point of view, elevated values of *D* indicate larger circulating spherical clusters. In the case of flowing rouleaux in normal subjects (likely rotating with the flow), a biased overestimation of the aggregate size is expected with this model. Consequently, the basic assumption behind the SFSAE imaging method did not affect the interpretation of results. To summarize, the SFSAE-based *in-vivo* measurements of RBC aggregation revealed that circulating diabetic RBC aggregates were bigger and presented a stronger resistance to shear rates compared to their counterpart in age-matched control subjects.

### Clinical perspectives

Hyper-aggregation of erythrocytes has been described in several pathologies, including HIV infection, myocardial infarction, sepsis, stroke, venous thrombosis and diabetes mellitus, to name a few examples [3]. However, it remains unclear whether it is caused by the pathology (through the action of inflammatory factors, oxidative stress agents or vascular alteration) or whether it contributes to the etiology of the pathology, and its associated vascular complications. Indeed, an increase in RBC aggregation may impede blood circulation and decreases tissue perfusion, subsequently sustaining the pathological process. The *in-vivo* hyper-aggregation observed in diabetic patients in both CEP and GSV might reflect some blood rheology disturbances in the upstream microvasculature. In diabetes mellitus, early hemorheological alterations have been linked to the development of vascular complications, like retinopathy, nephropathy, lower limb ischemia, brain ischemia, hypertension and atherosclerosis [11, 12, 29, 30]. A prospective clinical trial suggested that the level of RBC aggregation offers a high positive prediction for diabetic foot syndrome deterioration comparable to that associated with transcutaneous oxygen pressure, which is a widely used biomarker in clinical practice [11]. In light of these results, it was proposed to include hemorheological parameters in the screening of patients who presented a risk for foot ulceration, in order to obtain additional prognostic information. Because *ex-vivo* measurements of RBC aggregation require specific equipments that are unavailable in most clinical centers, the SFSAE cellular imaging technique, which can be implemented on a clinical ultrasound scanner, could represent a promising alternative to introduce rheological parameters in the management of diabetic patients. In this case, the targeted population would rather be patients in the early phase of diabetes mellitus development rather than patients with already confirmed vascular complications like in the present pilot study. Further larger scale and prospective studies have to be conducted in non-severe patients in order to address the predictive effectiveness of the proposed *in-vivo* RBC aggregation measurements.

### Limitations and summary

The SFSAE cellular imaging method allows reliable, non-invasive and *in-vivo* measurements of RBC aggregation using ultrasound. It has been conclusively used in preclinical animal studies on superficial or surgically exposed veins [17, 18]. According to the physics of ultrasound, biological tissue attenuation at 25 MHz limits measurements to superficial vessels (at approximately 1 cm deep or smaller). This may be seen as a limitation; nevertheless, because no differences in the RBC aggregation level were noticed between the cephalic and great saphenous veins for the studied population, measurements over the arm would be possible if severe edema precludes measurements over the patient’s feet. *In-vivo* hemorheological profile at the bedside thus seems feasible with the proposed imaging method.

Another limitation of the present study is that *D*_GSV_ only presented a trend (*p* = 0.055) for differences between groups. Specific flowing conditions could have impaired measurements in this foot area. Indeed, arteriovenous shunting has been proposed as a key factor of foot gangrene development in diabetic patients [26]. Such phenomena may have promoted unfavorable high shear flow conditions for the formation of RBC aggregates in this group. Moreover, a complex hemodynamic environment with several venous bifurcations was observed in our study and likely impacted RBC aggregate formation by disturbed flow at branching points, thus reducing *in-vivo* SFSAE measures of the aggregate size in diabetic patients. At the opposite, CEP segments were rectilinear, and could thus favor aggregate formation by inflammatory plasma proteins. Further studies will have to address this point further to clarify the measurement methodology.

To summarize, the present study confirmed for the first time the presence of larger RBC aggregates in diabetic patients under *in-vivo* flowing conditions. The SFSAE imaging method might be included in the management of diabetic patients to add prognostic information about the development of vascular complications. Further larger scale prospective studies would be required to address this latter point.
